# Effects of elevated extrinsic-stain firing temperatures on the optical, surface, and mechanical properties of lithium disilicate ceramics

**DOI:** 10.4317/jced.64313

**Published:** 2026-08-28

**Authors:** Matheus Morcela de Souza, Cássia Cunha de Lima, José Vitor Quinelli Mazaro, Natália Almeida Bastos Bitencourt, Letícia Moretti, Sandy Gabrielle Giorgetti Pereira, Débora Novais da Silva, Ana Carolina da Conceição Sanches, Marcella Santos Januzzi, André Luíz de Melo Moreno, Marcelo Coelho Goiato, Daniela Micheline dos Santos

**Affiliations:** 1 Department of Dental Materials and Prosthodontics, Araçatuba School of Dentistry, São Paulo State University (UNESP), Araçatuba, SP, Brazil; 2 University of Louisville,Louisville, Kentucky, USA; 3 College of Dental Medicine, Nova Southeastern University, Florida, USA

**Keywords:** Optical properties, extrinsic staining, surface roughness, microhardness, lithium disilicate.

## Abstract

**Background:**

This study evaluated the effect of different firing temperatures of extrinsic staining, including temperatures higher than those recommended by the manufacturer, on the optical properties, surface roughness, and microhardness of lithium disilicate ceramics.

**Materials and Methods:**

IPS e.max CAD blocks (Ivoclar) were sectioned and allocated into six groups (n=10) according to surface treatment: glaze only or glaze plus extrinsic staining, fired at 710°C, 770°C, or 820°C. A2 shade pigment paste (Ivoclar) was applied and fired according to manufacturer recommendations, followed by glazing at the same temperature. Color stability (ΔE00), translucency parameter (TP), and contrast ratio (CR) were assessed with a UV-visible spectrophotometer, surface roughness (Ra) was measured using contact profilometry and Vickers microhardness (MH) was obtained using a microdurometer. Data were analyzed with Shapiro-Wilk, Friedman or ANOVA, and Wilcoxon tests, with α=0.05.

**Results:**

For optical properties, firing at 770°C resulted in the lowest ΔE00 values, while 820°C yielded the highest TP values. CR differences before and after crystallization were significant in all groups except those fired at 820°C. Glazing significantly reduced surface roughness in all groups (p<0.001), with lower Ra values at 770°C and 820°C, independent of staining. Increased microhardness after stain+glaze was observed at 770°C.

**Conclusions:**

Firing at 770°C improved color stability and surface smoothness, while increased microhardness was observed when extrinsic stain and glaze were combined at this temperature. Firing temperature significantly influenced the optical properties and surface roughness of extrinsically stained lithium disilicate ceramics, with 770°C providing the most favorable balance between color stability and surface smoothness.

## Introduction

The demand for highly esthetic restorations that reproduce natural tooth characteristics-translucency, opacity, and color-has grown substantially. Dental ceramics are central to this goal because their optical, physical, and mechanical behavior depends on composition and processing; in general, a higher glass phase favors esthetics and translucency compared with a higher crystalline content [1,2]. Monolithic restorations have gained popularity for simplifying workflows and reducing veneer-related complications, while maintaining satisfactory wear resistance and durability under clinical surrogates [3,4]. 

Despite favorable intrinsic optics, many monolithic lithium disilicate restorations still require individualized characterization to achieve lifelike outcomes [5]. Extrinsic staining, applied after crystallization/sintering using low-fusing glass-based pigments, creates a thin, smooth surface layer that enhances optical appearance. However, color stability and surface effects may vary with ceramic composition and, critically, with the firing temperature used for the pigment and glaze [6]. Evidence indicates that repeated firings and temperature can alter color and surface topography, yet data remain limited and sometimes technique-specific [7].

Although several studies have investigated the influence of repeated firing cycles, glazing procedures, and thermal treatments on lithium disilicate ceramics [1,2,4,6,7], little attention has been given to extrinsic staining procedures used for the characterization of monolithic restorations. Extrinsic stains are routinely applied to reproduce natural tooth features and improve the esthetic integration of restorations [4]. In dental laboratory practice, firing temperatures higher than those recommended by manufacturers are sometimes used in an attempt to obtain improved gloss, translucency, and esthetic appearance. However, the effects of these empirically modified firing protocols on the optical, surface, and mechanical properties of lithium disilicate ceramics remain poorly understood [1,4,6,7].

Ceramic surface smoothness is clinically relevant: Smoother glazed or polished ceramics tend to retain less biofilm, reduce antagonist wear, and resist staining better than rougher surfaces [2-8]. Optical parameters, including color difference (ΔE_00_), translucency parameter (TP), and contrast ratio (CR), are essential to quantify esthetic performance and its clinical perceptibility/acceptability. Thresholds for ΔE_00_ and TP have been established for dental materials, supporting interpretation of laboratory findings in a clinically meaningful way [9-12]. Additionally, roughness metrics (e.g., Ra) provide standardized descriptors for surface quality, and microhardness is an indicator of resistance to deformation and surface wear [13].

Given these considerations, this *in vitro* study evaluated how different firing temperatures of extrinsic staining affect the surface roughness, microhardness, and optical properties of lithium disilicate ceramics, aiming to provide scientific evidence regarding firing protocols commonly used during the characterization of monolithic restorations. Glaze-only groups were included as controls to determine whether potential changes were associated with temperature alone or with the combined effect of extrinsic stain and glaze application. Therefore, this study tested the null hypothesis that different firing temperatures of extrinsic staining do not significantly affect the surface roughness, microhardness, or optical properties (color stability, translucency, and contrast ratio) of lithium disilicate ceramics.

## Materials and Methods

*Study Design*

This *in vitro* experimental study evaluated the influence of different firing temperatures (710°C, 770°C and 820°C) on extrinsic staining on the surface roughness, optical properties and microhardness of lithium disilicate ceramics. 

A priori power analysis was performed using G*Power software (version 3.1.9.7) for a two-tailed t-test comparing two independent means. The effect size (d=0.81) was calculated from previously published data [9] based on differences between means and standard deviations. With a significance level of α=0.05 and a desired statistical power of 90%, the minimum required sample size was determined to be seven specimens per group. To increase statistical reliability, ten specimens were included in each experimental group.

Pre-crystallized CAD/CAM lithium disilicate blocks (IPS e.max CAD MT A2; Ivoclar, Schaan, Liechtenstein) were sectioned, crystallized, and allocated into two main categories: Glaze only and extrinsic staining + glaze. Each category was divided into three groups according to the firing temperature: 710°C, 770°C, and 820°C (n=10 per group). Color (ΔE_00_), translucency parameter (TP), contrast ratio (CR), surface roughness (Ra), and microhardness (MH) were assessed after the firing process. The experimental design is illustrated in Figure 1.

*Sample Preparation*

Pre-crystallized lithium disilicate CAD/CAM blocks were sectioned using a low-speed diamond saw (Isomet 1000; Buehler, Lake Bluff, IL, USA) under water cooling to obtain rectangular specimens (12 × 18 × 2.0 mm). All the 60 specimens were made 25% larger than the final dimensions as indicated by ISO 6872:2015. Final dimensions were confirmed with a digital caliper (MPI/E-101; Mitutoyo, Tokyo, Japan). 

Crystallization was performed in a ceramic furnace (Programat P310 G2; Ivoclar) according to the manufacturer’s recommendations. Specimens were then randomly allocated into six experimental groups (n=10), according to the application of extrinsic staining (Ivoclar) and firing temperature: G710: Glaze only, fired at 710°C, G770: Glaze only, fired at 770°C, G820: Glaze only, fired at 820°C, PG710: Extrinsic staining + glaze, both fired at 710°C, PG770: Extrinsic staining + glaze, both fired at 770°C, and PG820: Extrinsic staining + glaze, both fired at 820°C (Figure 1).

*Extrinsic Staining Procedure*

One surface of each specimen in the PG groups was lightly moistened with IPS Ivocolor Mixing Liquid longlife (Ivoclar, Schaan, Liechtenstein) and coated with a thin uniform layer of IPS Ivocolor Shade Dentin A2 pigment paste (Ivoclar, Schaan, Liechtenstein) applied with a fine brush using parallel strokes. The specimens were fired in the furnace under the following cycle: Drying temperature of 403°C, closing time of 6 min, vacuum application, heating rate of 45°C/min until the target firing temperature, holding time of 1 min, followed by slow cooling inside the furnace. 

After staining (for PG groups) or directly after crystallization (for G groups), glaze application (IPS Ivocolor Glaze; Ivoclar) was performed, followed by firing at the same temperature used for the staining cycle.

*Optical Properties Measurement*

Optical properties were measured with a UV-visible reflection spectrophotometer (UV-2450; Shimadzu, Kyoto, Japan) using the CIE Lab* color space with a D65 illuminant and a 2° observer angle, in the wavelength range of 380-780nm and a measurement aperture of 10 mm. 

Color difference (ΔE_00_) was calculated using the CIEDE2000 formula. Thresholds for perceptibility (0.81) and acceptability (1.77) were applied. Translucency parameter (TP) was calculated from measurements taken over black and white backgrounds, with ΔTP_00_ thresholds of 0.62 (perceptibility) and 2.62 (acceptability). Contrast ratio (CR) was calculated from the reflectance over black and white backgrounds, higher CR values indicate greater opacity. 

Measurements were performed at two time: After crystallization and after glaze firing with or without extrinsic staining.

- Surface Roughness and Microhardness Measurement

Surface roughness was assessed using a contact profilometer (SJ-401; Mitutoyo) equipped with a 2 μm diamond stylus. The cut-off value (λc) was 0.08 mm, and the stylus speed was 0.05 mm/s. For each specimen, three parallel measurements were taken, and the mean values of Ra (average roughness) were recorded. 

Vickers microhardness was analyzed with a microhardness tester (HMV-2Tl Shimadzu - Kyoto, Japan), which had been calibrated with 2.92N for 10 seconds. The average of three readings performed on the disc surface was calculated. The Vickers microhardness was calculated with the test machine software program from the mean length of the diagonals of the indentations.

*Statistical Analysis*

Data normality and homoscedasticity were assessed using the Shapiro-Wilk and Levene tests, respectively. Surface roughness data (Ra) showed non-normal distribution and were analyzed using the Friedman test followed by the Durbin-Conover post hoc test. Optical properties (ΔE_00_, TP, and CR) showed normal distribution and were analyzed using one-way or two-way ANOVA, followed by Tukey’s post hoc test. Microhardness data were analyzed using the Kruskal-Wallis test followed by pairwise Wilcoxon tests for intragroup comparisons. The significance level was set at α=0.05. All analyses were performed using Jamovi software (version 2.3; The Jamovi Project, Sydney, Australia).

## Results

For the surface roughness (Ra, µm) results are presented in Table 1 and Figure 2. There was a significant reduction in surface roughness after glazing (T1 vs T0) in all groups, according to Friedman’s test (*p<*0.001) and Durbin-Conover post hoc comparisons (*p<*0.001). Within the G (glaze only) condition, Ra values were significantly lower at 770°C and 820°C compared with 710°C (*p<*0.05). The same was observed for PG (glaze + pigment) condition, with significantly lower Ra at 770°C and 820°C compared with 710°C (*p<*0.05).

For the color change values (∆E00), the one-way-ANOVA indicated a statistically significant difference in the heat treatment of the vitrified samples, both without extrinsic pigment (*p=*0.001) and with extrinsic pigment (*p=*0.005), with the samples crystallized at a temperature of 770 ºC showing the lowest color change values (Table 2 and Figure 3A).

For the translucency values (TP), the two-way-ANOVA indicated a statistically significant difference in the interaction of the heat treatment and time factors for the glazed samples without (*p=*0.026) and with extrinsic pigment (*p=*0.011), with the samples heat-treated at a temperature of 820 ºC showing the highest translucency values after the crystallization process (Table 2 and Figure 3B).

For the degree of contrast values, the two-way ANOVA indicated a statistically significant difference in the interaction between the heat treatment and time factors for the samples glazed without pigment (*p<*0.001); and a statistically significant difference in the time factor for the samples glazed with pigment (*p<*0.001). With the exception of the samples heat-treated at a temperature of 820°C, all other samples showed a statistically significant difference in the degree of contrast values before and after the crystallization process (Table 2 and Figure 3C). 

Table 3 presents the microhardness values (median (Q1-Q3)) for each group at the two evaluation times (T0 and T1). Kruskal-Wallis analysis showed no statistically significant differences among firing temperatures within each treatment (G and PG) at either T0 or T1 (*p>* 0.05). In the intragroup comparison (Wilcoxon test), a significant reduction in microhardness was observed in groups G770 (*p=*0.019) and G820 (*p=*0.019) after glaze application. Group PG770 showed a significant increase in microhardness after stain+glaze (*p=*0.049). No significant differences between times were found for the other groups.

## Discussion

This study tested the null hypothesis that the firing temperature of the extrinsic stain and glaze would not influence the optical properties, surface roughness, and microhardness of lithium disilicate ceramics. The results led to the rejection of this hypothesis, as firing temperature significantly influenced several of the evaluated outcomes. In terms of optical behavior, 770°C resulted in the lowest ΔE_00_ values, whereas 820°C yielded the highest translucency parameter (TP) values, with contrast ratio (CR) varying according to the measurement stage and firing protocol. Glazing substantially reduced Ra values in all groups, while firing at 770°C and 820°C produced smoother surfaces compared with 710°C.

Our findings align with recent evidence [2,6,8] demonstrating that multiple firings and variations in thermal processing can alter the color, translucency, and surface features of glass-based ceramics. While previous studies have primarily focused on repeated firing cycles, glazing procedures, or their effects on the optical and mechanical properties of lithium disilicate ceramics, the present investigation specifically addressed extrinsic staining protocols applied to monolithic restorations and evaluated firing temperatures frequently used in dental laboratory practice but not recommended by the manufacturer.

Increasing the number of firing cycles in Virgilite-containing lithium disilicate ceramics led to significantly higher color change (ΔE_00_), reduced translucency (TP_00_), especially in thinner specimens, and decreased biaxial flexural strength, underscoring the risks of altering firing protocols [4]. In contrast, in the present study, specimens subjected to two (crystallization + glaze) or three firings (crystallization + extrinsic stain + glaze) did not exhibit deterioration of surface or optical properties; in some cases, additional firings even improved surface smoothness or translucency [1]. Although our experiment was not primarily designed to investigate the effect of the number of firings, this difference suggests that the impact of repeated firings may depend on ceramic formulation, pigment/glaze composition, and firing parameters.

Beyond individual experiments, a recent systematic review highlighted that composition (lithium disilicate, zirconia-reinforced lithium silicate, advanced lithium disilicate) and manufacturing technique (press vs CAD/CAM) distinctly influence mechanical and optical properties [5]. These findings provide important background for interpreting the variability in the literature regarding firing effects. Moreover, glazing and specific thermal treatments may ameliorate surface defects through microstructural reorganization [6], although multiple firing cycles can affect hardness, fracture toughness, and surface roughness in lithium disilicate ceramics [7].

Our results reveal that in glaze-only groups, only firing at 770°C yielded ΔE_00_ values below the perceptibility threshold, indicating optimal color stability, according to the perceptibility (0.81) and acceptability (1.77) thresholds reported in the literature [11]. Firing at 710°C and 820°C resulted in perceptible but clinically acceptable changes. In pigment + glaze groups, all firing temperatures produced ΔE_00_ values well above the acceptability limit; however, this outcome was expected, as extrinsic staining is intended to produce a deliberate color shift relative to the crystallized A2 substrate. Thus, higher ΔE_00_ values in these groups reflect the intended characterization rather than degradation. Within stained specimens, firing at 770°C yielded the lowest ΔE_00_ among temperatures, indicating comparatively better chromatic stability when extrinsic staining is used. Nevertheless, future studies should investigate the long-term stability of these pigments under simulated clinical conditions, including exposure to staining beverages and foods, prophylactic pastes, mouthrinses, and toothbrushing, to determine whether color changes occur over time or if pigment loss affects the esthetic outcome.

Reviews and experimental studies [9,10] emphasize that TP is sensitive to thickness, composition, and firing parameters, with changes in the glass/crystalline phase ratio directly influencing light scattering and absorption. In our study, firing at 820°C led to increased translucency parameters, indicating that elevated firing temperatures may favor microstructural redistribution of the glass-ceramic phase, improving light passage through the material. This observation is consistent with prior reports demonstrating that translucency in lithium disilicate ceramics can be controlled by the firing temperature and thermal protocol [14].

Regarding surface roughness, previous studies have reported that glazing technique and firing conditions impact the final topography, that presents intercrystallite defects caused by the milling process, with most protocols reducing roughness compared with the pre-glazed state [6,8], consistent with our results at T1.

The absence of significant differences in microhardness among firing temperatures within the groups suggests that variations between 710°C, 770°C, and 820°C did not substantially influence the surface mechanical response of lithium disilicate ceramics. However, intragroup analysis revealed distinct effects according to surface treatment. Glaze-only groups showed significant reductions in microhardness at 770°C and 820°C, whereas a significant increase was observed only in PG770 after the stain + glaze procedure. These findings suggest that the influence of post-crystallization treatments on surface hardness may depend on the interaction between firing temperature and the presence of extrinsic stain.

The reduction in microhardness observed in groups G770 and G820 after glaze firing may be associated with the predominantly glassy nature of the glaze layer, which can reduce overall surface hardness [5,14]. Conversely, the increase observed in PG770 may indicate that the interaction between stain and glaze at this temperature promoted changes in the surface layer that increased hardness. However, because no microstructural analyses were performed, this interpretation should be considered speculative. These findings reinforce that surface treatment may exert a greater influence on surface mechanical properties than moderate variations in firing temperature.

- Possible mechanisms

The differences observed among firing temperatures may be associated with microstructural changes induced by thermal processing. Higher firing temperatures can enhance the flow of the glassy phase, promoting surface leveling and reducing superficial irregularities, which may explain the lower roughness values observed at 770°C and 820°C. Similar effects have been reported for lithium disilicate ceramics subjected to glazing and repeated firing procedures [6,8,13].

In addition, thermal treatments may influence light scattering within the ceramic structure, contributing to the differences observed in translucency and contrast ratio. Previous studies have shown that firing protocols can modify the optical behavior of lithium disilicate ceramics without compromising their mechanical performance [6].

Color differences may also be related to temperature-dependent interactions between extrinsic stains and the ceramic surface. However, since no microstructural or chemical analyses were performed, these mechanisms should be interpreted as possible explanations for the observed optical changes [3,4].

Manufacturer’s recommendation vs. laboratory practice

The manufacturer recommends a firing temperature of 710°C for extrinsic staining and glazing of lithium disilicate ceramics. However, higher temperatures are sometimes used during laboratory characterization procedures in an attempt to improve esthetic outcomes. In the present study, increasing the firing temperature influenced the optical and surface properties of the material, with 820°C resulting in higher translucency and 770°C providing lower color change and smoother surfaces. These findings suggest that firing temperature selection may affect the final esthetic performance of monolithic lithium disilicate restorations and should be carefully considered during laboratory processing.

*Clinical implications*

From a clinical perspective, the present findings may help guide the selection of firing temperatures according to the desired esthetic outcome. Firing at 770°C was associated with lower color change (ΔE_00_) values and smoother glazed surfaces, whereas firing at 820°C resulted in higher translucency values. Therefore, different firing temperatures may be considered depending on the esthetic characteristics desired for monolithic lithium disilicate restorations.

These findings should be interpreted in conjunction with other factors known to influence the performance of ceramic restorations, including material thickness and firing protocols, which may affect optical and mechanical behavior [8,9]. Within the limitations of this study, the results suggest that firing temperature selection can influence the balance between esthetics and surface characteristics of lithium disilicate restorations.

*Limitations*

This was an *in vitro* study conducted using a single lithium disilicate ceramic system, one extrinsic stain shade, and standardized specimen thickness. Long-term chemical and mechanical aging, which may further influence color stability, surface characteristics, and microhardness, was not evaluated. In addition, the findings may not be directly extrapolated to other lithium disilicate formulations, staining systems, or clinical situations involving different restoration thicknesses. Future studies should incorporate multifactorial aging protocols, evaluate different staining materials and ceramic systems, and assess clinically relevant restoration geometries.

## Conclusions

Firing temperature directly influenced both the optical performance and surface roughness of lithium disilicate ceramics subjected to extrinsic staining. Firing at 770°C yielded the least color alteration and smoothest surfaces, while firing at 820°C enhanced translucency. Nevertheless, a significant increase in microhardness was observed only when the pigment and glaze were applied in combination. Clinically, these findings suggest that the stain + glaze protocol at optimized firing temperatures may provide a potentially effective approach to achieving a balance between esthetic and mechanical performance in lithium disilicate restorations.

## Figures and Tables

**Table 1:** Surface roughness (Ra, µm) for each firing temperature and timepoint (T0 and T1).

**Group**	**T0 (median (Q1-Q3)**	**T1 (median (Q1-Q3)**
G710	0.427 (0.291-0.808)ᵃ*	0.158 (0.095-0.242)ᵃ*
G770	0.344 (0.262-0.864)^a^*	0.048 (0.034-0.143)ᵇ*
G820	0.599 (0.354-0.802)^a^*	0.075 (0.044-0.132)ᵇ*
PG710	0.384 (0.233-0.638)ᵃ*	0.362 (0.286-0.409)ᵃ*
PG770	0.361 (0.306-0.547)^a^*	0.115 (0.091-0.168)ᵇ*
PG820	0.369 (0.279-0.593)^a^*	0.078 (0.053-0.144)ᵇ*

Different superscript lowercase letters indicate significant differences among firing temperatures within the same group and timepoint (p<0.05, Durbin-Conover post hoc test). Asterisks indicate significant differences between T0 and T1 within the same group and temperature (p<0.05, Friedman’s test).

**Table 2:** Optical properties (mean±SD) for each firing temperature and timepoint.

**Group**	**ΔE**_00_ **​**	**TP - Before**	**TP - After**	**CR - Before**	**CR - After**
G710	1.06 ± 0.45ᵃ	17.9 ± 1.42ᴬᵃ	15.9 ± 1.33ᴮᵃ	0.60 ± 0.03ᴬᵃ	0.65 ± 0.03ᴮᵃ
G770	0.59 ± 0.26ᵇ	17.1 ± 1.40ᴬᵃ	14.6 ± 1.39ᴮᵃ	0.62 ± 0.03ᴬᵃ	0.68 ± 0.04ᴮᵃ
G820	0.95 ± 0.92ᵃᵇ	16.6 ± 3.75ᴬᵃ	16.0 ± 1.14ᴬᵃ	0.61 ± 0.03ᴬᵃ	0.65 ± 0.03ᴮᵃ
PG710	4.33 ± 0.83ᵃᵇ	17.5 ± 1.38ᴬᵃ	15.6 ± 1.14ᴮ^a^	0.61 ± 0.03ᴬᵃ	0.66 ± 0.03ᴮᵃ
PG770	3.95 ± 0.58ᵇ	17.0 ± 0.85ᴬᵃ	16.9 ± 0.90ᴬ^a^	0.61 ± 0.02ᴬᵃ	0.62 ± 0.02ᴬᵃ
PG820	4.69 ± 0.72ᵃ	17.2 ± 1.05ᴬᵃ	15.8 ± 1.34ᴮ^a^	0.62 ± 0.02ᴬᵃ	0.67 ± 0.13ᴮᵃ

Different superscript lowercase letters indicate significant differences among firing temperatures within the same group and timepoint (p<0.05). Different uppercase letters indicate significant differences between before and after firing within the same group and temperature (p<0.05).

**Table 3:** Microhardness, median (Q1-Q3), for each group at T0 and T1.

**Group**	**T0**	**T1**
G710	595.2 (553.0-616.8)	567.5 (562.8-583.0)
G770	584.2 (554.2-598.5)	527.5 (510.1-555.8)*
G820	606.7 (564.8-619.5)	544.5 (541.4-556.1)*
PG710	552.2 (542.5-568.7)	574.7 (570.5-578.5)
PG770	562.8 (555.0-576.6)	590.2 (578.2-598.2)*
PG820	569.2 (552.2-579.2)	584.3 (562.0-591.1)

*Significant difference between T0 and T1 within the same group (Wilcoxon test, p<0.05).

**Figure 1:**
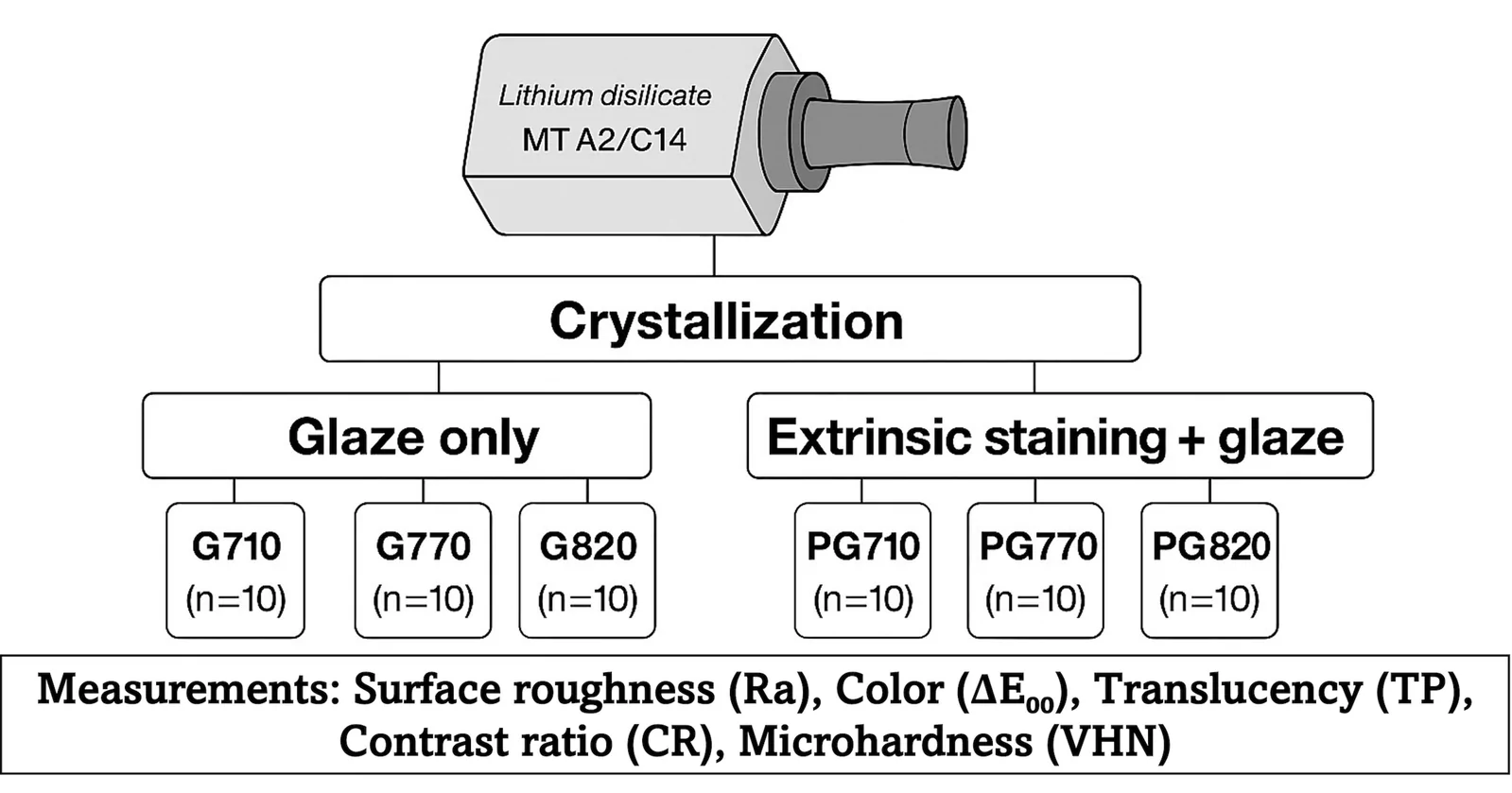
Experimental design. Lithium disilicate CAD blocks were crystallized and allocated into two categories: glaze only and extrinsic staining + glaze. Each category was divided into three groups according to firing temperature (710°C, 770°C, and 820°C) (n=10 per group). Surface roughness (Ra), color difference (ΔE_00_), translucency parameter (TP), contrast ratio (CR) and microhardness (VHN) were measured after firing.

**Figure 2:**
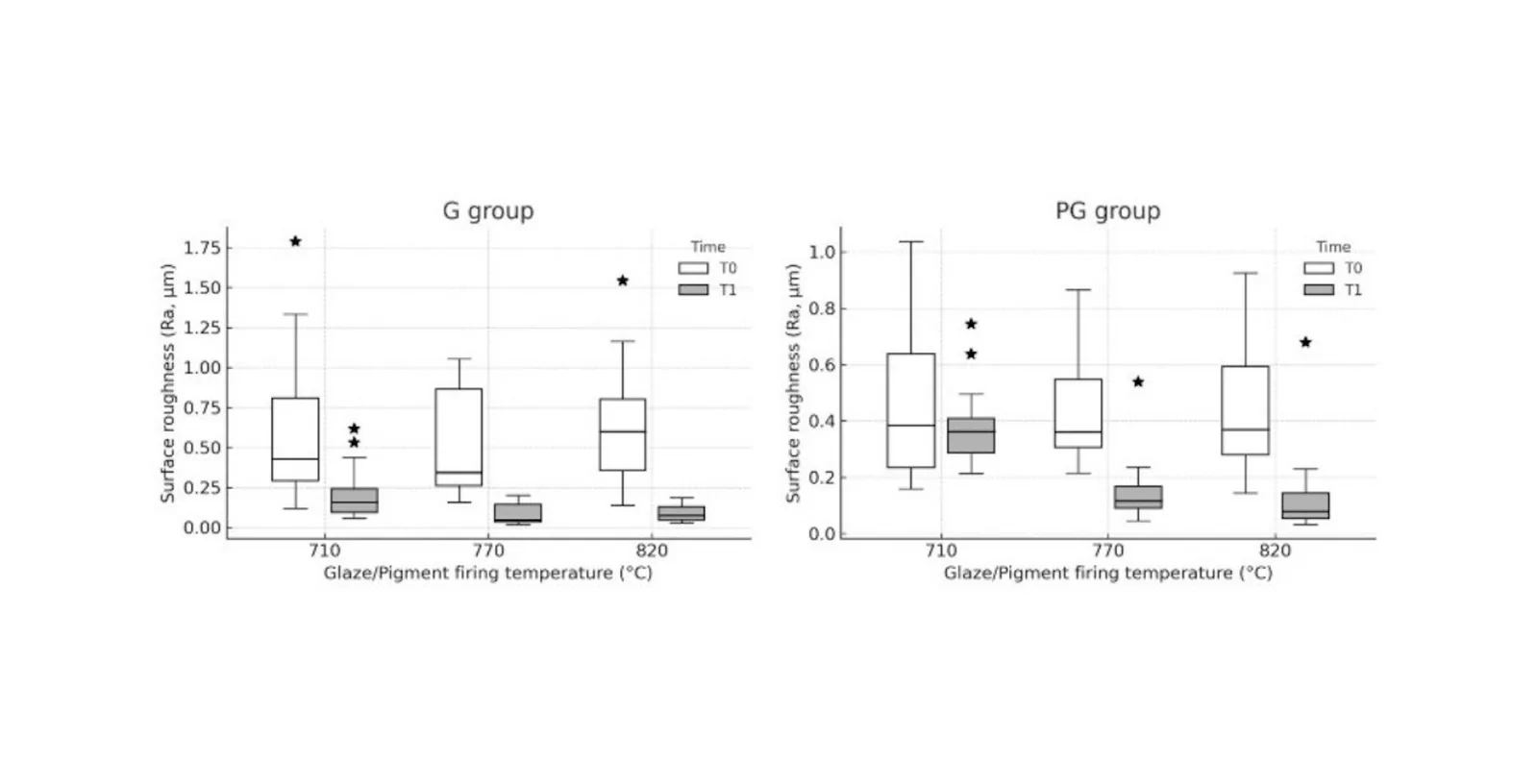
Box plot of surface roughness (Ra, μm) before (T0) and after (T1) glazing for each glaze/pigment firing temperature within the G (glaze only) and PG (glaze + pigment) groups. The central line represents the median, the box represents the interquartile range (Q1-Q3), and the whiskers indicate the minimum and maximum values excluding outliers (★). Statistical analysis was performed using Friedman’s test followed by Durbin-Conover post hoc comparisons (p<0.05).

**Figure 3:**
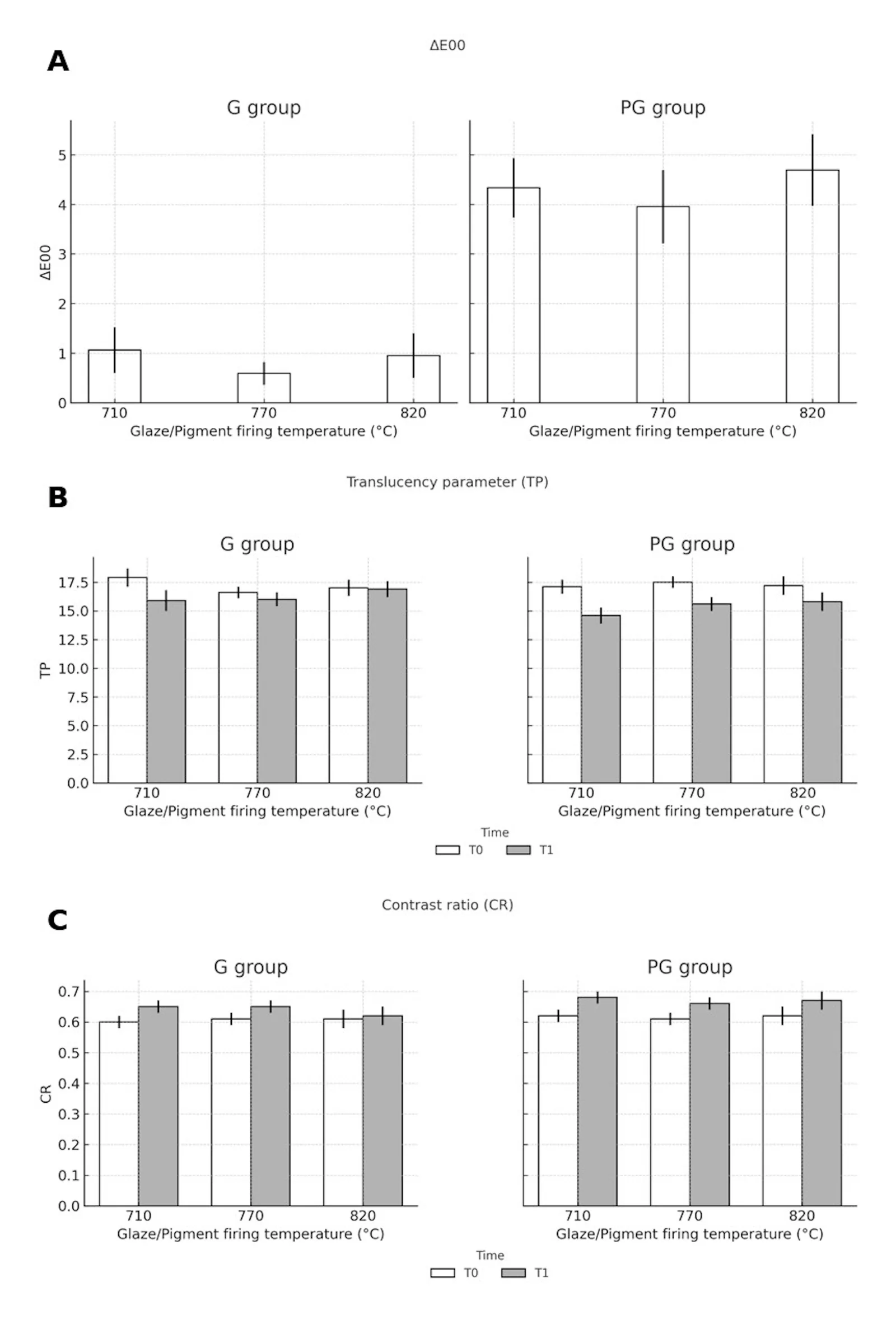
Mean (± standard deviation) values for (A) color change (ΔE_00_), (B) translucency parameter (TP), and (C) contrast ratio (CR) before (T0) and after (T1) glazing for each glaze/pigment firing temperature within the G (glaze only) and PG (glaze + pigment) groups. Statistical analysis: one-way ANOVA for ΔE_00_, and two-way ANOVA for TP and CR (p<0.05).

## Data Availability

Declared none.
